# Isothermal Adsorption Properties for the Adsorption and Removal of Reactive Blue 221 Dye from Aqueous Solutions by Cross-Linked β-Chitosan Glycan as Acid-Resistant Adsorbent

**DOI:** 10.3390/polym10121328

**Published:** 2018-11-30

**Authors:** Chih-Wei Chiu, Ming-Tsung Wu, Jimmy Chi-Min Lee, Ting-Yu Cheng

**Affiliations:** 1 Department of Materials Science and Engineering, National Taiwan University of Science and Technology, Taipei 10607, Taiwan; d10404011@mail.ntust.edu.tw (M.-T.W.); terri4020010@gmail.com (T.-Y.C.); 2 Clean Instruments Co., Ltd., New Taipei City 24301, Taiwan; jimmy@cleaninst.com

**Keywords:** chitosan, adsorption, reactive blue 221, dye, acid-resistant property, effluent treatment

## Abstract

Dye effluent causes serious pollution and damage to the environment and needs a series of treatments before it can be discharged. Among the numerous effluent treatment methods, adsorption is the simplest and does not cause secondary pollution. Bio-adsorbents are especially advantageous in the treatment of low-concentration dye effluent. In this study, the adsorption and removal capacities of unmodified α- and β-chitosan and modified β-chitosan (β-chitosan cross-linked with triethylenetetramine, BCCT) on C.I. Reactive Blue 221 (RB221) dye were compared. The experiments were performed on the adsorption of the RB221 dye by unmodified α- and β-chitosan and cross-linkage–modified BCCT at different temperatures and for different durations, which are presented along with the relevant adsorption kinetics calculations. According to the results, as the temperature increased from 303 to 333 K, the initial adsorption rates of the adsorbents, α-chitosan, β-chitosan, and BCCT, for the RB221 dye, changed from 1.01 × 10^2^, 4.74 × 10^2^, and 1.48 × 10^6^ mg/g min to 5.98 × 10^4^, 4.23 × 10^8^, and 1.52 × 10^13^ mg/g min, respectively. BCCT thus showed the best adsorption for the dye at all temperatures from the Elovich model. These results confirmed the successful introduction of a polyaminated and cross-linked extended structure as a modification for the BCCT adsorbent, which makes it resistant to acid hydrolysis and gives it the functional amine group for dye adsorption, thereby promoting the ability of BCCT to adsorb dyes under strongly acidic conditions. The compound synthesized in this study is expected to be a good choice in the future for purifying strongly acidic effluent containing anionic organic dyes.

## 1. Introduction

With the advancement of human civilization, color has become an indispensable element in the pursuit of fashion and aesthetics. This, however, has also generated new environmental issues. Dye effluent is derived from a variety of economic activities and production processes. The main sources of such pollution include textile dyeing and finishing [1,2,3], printing and dyeing [4], and leather processing [5], all of which involve the use of dyes for coloring to eventually fabricate various kinds of end products [6,7]. More than 10,000 types of commercial synthetic dyes are used each year in the coloring step of various manufacturing processes. Reactive dyes account for about 50% of the total annual consumption of all dyes, with a total production output of more than 7 × 10^5^ tons on the global market. About 5%–10% of dyes produce industrial effluent during the dyeing process [8,9,10]. The dyes in dye effluent are easily detected by the naked eye even at trace concentrations, which affect the appearance and transparency of water. Generally speaking, most dyes are ecologically toxic, causing mutations and possibly cancers in organisms. Dye effluent enters surface water and groundwater in different ways, damaging the ecological environment and posing threats to human health. Belpaire et al. analyzed the water quality of Belgian rivers and found residues of sixteen types of dyes and their metabolites in the tissue samples of wild European eels. About 77% of these eels were contaminated by dyes, and 25%–58% of the muscle tissues sampled contained malachite green and crystal violet dyes [11]. Long-time workers in the dye industry may suffer from dye-induced cancers [12] and other serious health problems, such as renal dysfunction and damage to the reproductive system, liver, brain, and central nervous system [13,14]. Therefore, the removal of dyes from industrial effluent before its discharge into the ecological system is very important, both as an environmental problem and a topic of health research.

In the literature, several methods have been reported for purifying industrial waste and reducing its environmental damage. The treatment of dye effluent could be achieved generally in three ways: (1) chemical treatment, which breaks down the dye molecules chemically and removes them from the dye effluent [15]; (2) physical treatment, which removes various dyes by physisorption of the adsorbents [16]; and (3) biological treatment, a effluent treatment method that is achieved through anaerobic biological methods that utilize facultative, as well as specific, anaerobic bacteria to degrade macromolecular organic compounds into simple molecular compounds and then into methane and carbon dioxide [17,18]. Physical treatment is superior to the other two dye effluent removal technologies because of its simple operation and high application efficiency. Recent studies have found that adsorbent materials can be used for effluent treatment and water purification. These adsorbent materials, including polysaccharides, cellulose, and chitin, are mainly used for the recycling of agricultural and fishery waste. Chitosan can be obtained by chemical treatment. Because the structure of chitosan has amine groups and hydroxyl groups, it can be used as a chelation point of adsorption material. It is noteworthy that dye adsorption may involve different mechanisms (chelation and electrostatic attraction) depending on the composition of the solution, pH value, and the chemical structure and morphology of the dye [10].

Recently, an important issue to be resolved for effluent treatment has been to find various biosorbents, such as lignin [19], chitosan [20], and cellulose–chitosan composites [21], which can effectively remove organic dyes. In recent studies, chitosan has been shown to have a good affinity for many types of dyes. The mechanism of dye removal and the future impacts of chitin, chitosan, and chitosan derivatives have been investigated [22]. Chitosan adsorbs acidic dyes by electrostatic attraction, as the protonated amine groups in acidic aqueous solutions are cationic. At the same time, chitosan is also soluble in acidic media and cannot be used as an insoluble adsorbent. The removal of acidic dyes from acidic effluent could be realized by preparing chitosan with good adsorption performance [23] and stability in low-pH solutions via chemical grafting modifications [24]. A review of the literature shows that α-chitosan from crab and prawn shells is the most frequently used for chemical modification by grafting. In this study, β-chitosan from squid gladius was used as the raw material on which the chemical modification was carried out. Functional group protection was performed on the amine group of the chitosan C2 by treating it with benzaldehyde to form a Schiff base. Grafting and cross-linking were done by reacting chitosan with epichlorohydrin and triethylenetetramine [25]. The effects of pH, adsorption temperature, adsorption duration, and initial dye concentration on the adsorption performance of α-chitosan and modified and unmodified β-chitosan on Reactive Blue 221 (RB221) dye are discussed. Lastly, the adsorption kinetics, adsorption isotherm, and adsorption mechanism of modified β-chitosan for the RB221 dye are analyzed.

## 2. Materials and Methods

### 2.1. Materials

**Figure 1:**
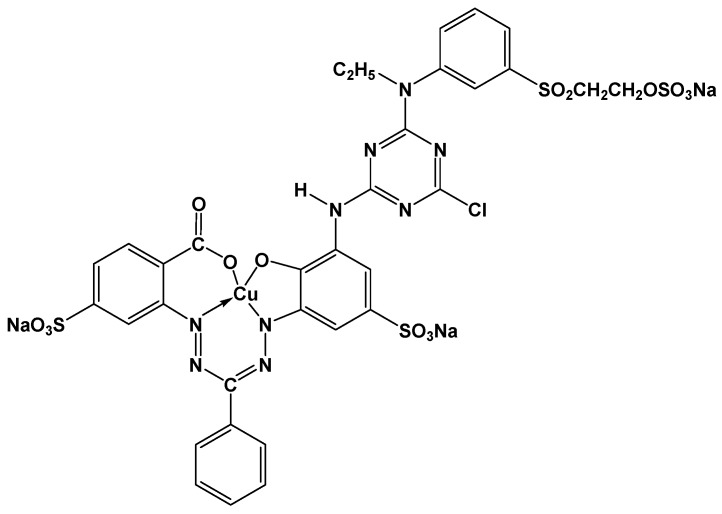
Chemical structure of the C.I. Reactive Blue 221 (RB221) dye.

90% deacetylated food-grade α- and β-chitosan, both of molecular weight 500 KD, were purchased from Charming and Beauty Co., Taipei, Taiwan. Reagent-grade benzaldehyde was purchased from TEDIA, Fairfield, OH, USA. Reagent-grade 99% epichlorohydrin was purchased from Alfa Aesar, Tewksbury, MA, USA. Reagent-grade 60% triethylene-tetramine and methanol were purchased from ACROS, NJ, USA. Reagent-grade 95% ethanol and ethyl ether were purchased from Shimakyu Chemical Co., Ltd., (Osaka, Japan). Reagent-grade 37% hydrochloric acid was purchased from SCHARLAU, Barcelona, Spain. Reagent-grade dimethyl sulfoxide was purchased from MERCK, Darmstadt, Germany. Technical-grade C.I. Reactive Blue 221 dye of molecular weight 890 g/mol was purchased from NIPPON KAYAKU Co., Ltd. (Tokyo, Japan) The chemical structure of this compound is shown in Figure 1.

### 2.2. Synthesis of β-Chitosan Cross-Linked with Triethylenetetramine (BCCT)

**Figure 2:**
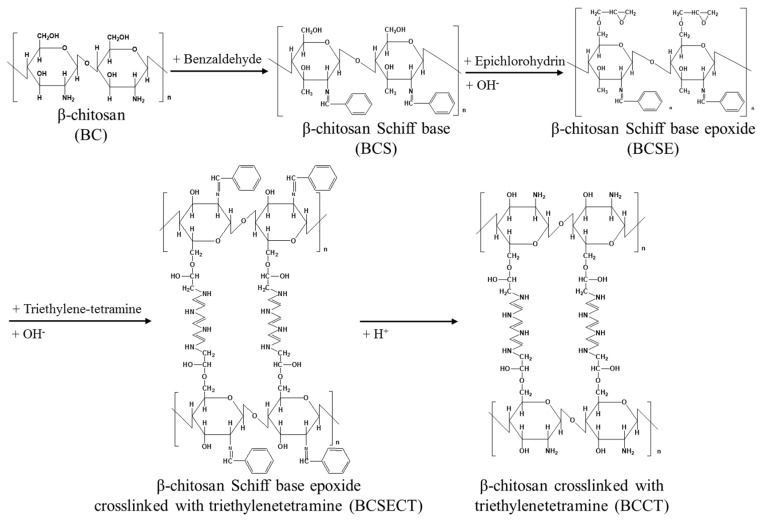
Synthesis route of the modified β-chitosan (i.e., β-chitosan cross-linked with triethylenetetramine (BCCT)) and its intermediate products.

The synthesis of β-chitosan cross-linked with triethylenetetramine was done as in our previous work [25]. The modified β-chitosan derivative and its intermediates were formed as shown in the reaction in Figure 2. Their structures were determined by Fourier-transform infrared spectra (FTIR) and solid-state nuclear magnetic resonance spectroscopy (NMR) and are shown in Figures S1 and S2, respectively. β-chitosan powder (BC) and benzaldehyde in a ratio of 1 g: 5 mL were firstly reacted for 3 h at 60 °C. The products were washed a few times with an ethanol/deionized water solution and vacuum filtered. They were purified with ether in a Soxhlet extractor for 12 h and dried at 90 °C for 12 h to obtain the β-chitosan Schiff base (BCS). The BCS and epichlorohydrin in a ratio of 1 g: 5 mL and 100 mL of an alkali solution (concentration 0.4 mol/L) were added into the reactor and reacted at 65 °C for 6 h to prepare the β-chitosan Schiff base epoxide (BCSE). The products were filtered and washed a few times with an ethanol/deionized water solution to remove any unreacted chemicals and dried at 90 °C for 12 h to obtain the BCSE. Next, the BCSE and triethylenetetramine in a ratio of 1 g: 3 mL were put into the reactor with 100 mL of an alkali solution (concentration 0.1 mol/L) at 65 °C for 6 h to perform the grafting between the BCSE and triethylenetetramine. The products were vacuum filtered and washed a number of times with an ethanol/deionized water solution to remove any unreacted chemicals and dried at 90 °C for 12 h to obtain the ß-chitosan Schiff base epoxide cross-linked triethylenetetramine (BCSECT). Next, 1 N hydrochloric acid solution was added to BCSECT at room temperature and stirred for 2 h, followed by filtering and repeated washing with an ethanol/deionized water solution to remove any unreacted chemicals. The products were then transferred to a 1 N NaOH solution to alkalize for 2 h, filtered, and washed with deionized water until they were neutral. They were dried at a constant temperature of 90 °C for 12 h to obtain β-chitosan cross-linked with triethylenetetramine (BCCT) as the final product.

### 2.3. Adsorption of the Reactive Dye

The adsorption of the adsorbent for the RB221 dyes was tested under different conditions, such as different pH, adsorption temperature, adsorption duration, and initial dye concentration.

The influence of different pH (pH range of 2–12) on the adsorption capacity of the adsorbent was tested. First, 25 mL of the RB221 dye solution (concentration 600 mg//L) and 0.25 g adsorbent were added to the reaction flask and stirred with a magnetic stirring bar at a speed of 800 rpm. After 30 min, the adsorbent was separated from the aqueous solution by centrifugation at a centrifuge speed of 6000 rpm for 15 min. The supernatant was extracted to analyze the final residual concentration.

The adsorption isotherms of the adsorbent were also determined at different adsorption temperatures (303, 313, and 323 K). First 25 mL of the RB221 dye solution with different initial concentrations (1000, 1400, 1800, 2200, 2600, and 3000 mg/L, at pH 5.0) was added to the reaction flask, followed by 0.25 g of the adsorbent. The mixture was stirred at 800 rpm for 24 h with a magnetic stirring bar. The adsorbent was separated from the aqueous solution by centrifugation at 6000 rpm for 15 min. The supernatant was extracted to analyze the concentration.

Next, the adsorption kinetics of the adsorbent were measured at different adsorption temperatures (303, 318, and 333 K). First, 25 mL of the RB221 dye solution (at a concentration of 1000 mg/L and pH 5.0) was added to the reaction flask, followed by 0.25 g of the adsorbent. The mixture was stirred at 800 rpm for different durations (15, 30, 45, 60, 90, 120, 150, and 180 min) with a magnetic stirring bar. The adsorbent was separated from the aqueous solution by centrifugation at 6000 rpm for 15 min. The supernatant was extracted to analyze the concentration.

The concentration of each extracted supernatant was determined by UV–visible spectroscopy (V-630 mode Spectrophotometer, JASCO CORPORATION, Tokyo, Japan). The sorption capacities of the adsorbent at a given time and at the state of equilibrium were calculated by Equations (1) and (2), respectively [26]:

(1) $$q_{e}=\frac{(C_{0}-C_{e})•V}{m}$$

(2) $$q_{t}=\frac{(C_{0}-C_{t})•V}{m}$$

where *q_e_* is the amount of the RB221 dye adsorbed at the state of equilibrium (mg/g), *q_t_* is the amount of the RB221 dye adsorbed at the adsorption time t (mg/g), *C*_0_ is the initial concentration of the RB221 dye in the liquid phase (mg/L), *C*_e_ is the concentration of the RB221 dye remaining in the liquid phase (mg/L), *C_t_* is the concentration of the RB221 dye in the liquid phase at the adsorption time t (mg/L), *V* is the volume of the RB221 dye solution (L), and *m* is the mass of the adsorbent used (g).

### 2.4. Characterization and Measurements

#### 2.4.1. Fourier-Transform Infrared Spectra (FTIR) Were Collected on Perkin Elmer, Waltham, MA, USA

The powdered sample was dried and dehydrated in an oven at 100 °C for 24 h and ground in an agate mortar. KBr powder of IR grade was added and ground together until the mixture was of an even consistency. The paste was pressed into a pellet, on which FTIR spectroscopy was run 32 times.

#### 2.4.2. Ultraviolet–Visible (UV–VIS) Data Were Collected on V-670 Mode Spectrophotometer

The powdered sample was dried and dehydrated in an oven at 100 °C for 24 h. A certain amount of the sample was filled and pressed into the sample holder. For liquid samples, a certain volume of the sample was drawn and added into the cell. In both the cases, UV absorption spectroscopy was performed 32 times.

#### 2.4.3. Zeta Potential Analysis Was Conducted on Malvern Instruments Zetasizer Nano ZS90, Westborough, MA, USA

A total of 10 mg of the sample was mixed well with 20 mL deionized water. The measurement was performed in Zeta cells.

#### 2.4.4. Elemental Analysis (EA) Was Conducted on Thermo Flash 2000, Waltham, MA, USA

The sample to be measured was firstly dried and dehydrated in an oven at 100 °C for 24 h. About 3–5 mg of the sample was placed in a tin capsule and folded into small cubes that were arranged on the automated sampler for analysis.

#### 2.4.5. Solid-State Nuclear Magnetic Resonance Spectroscopy Was Performed on Varian Inova AS500 MHz, Palo Alto, CA, USA 

The sample to be measured was firstly dried and dehydrated in an oven at 100 °C for 24 h and ground into a fine powder in an agate mortar. It was put into a sample tube and subjected to ^13^C NMR under cross-polarization/magic angle spinning.

## 3. Results and Discussion

### 3.1. Effect of pH on the Adsorption Capability of the Various Adsorbents

**Figure 3:**
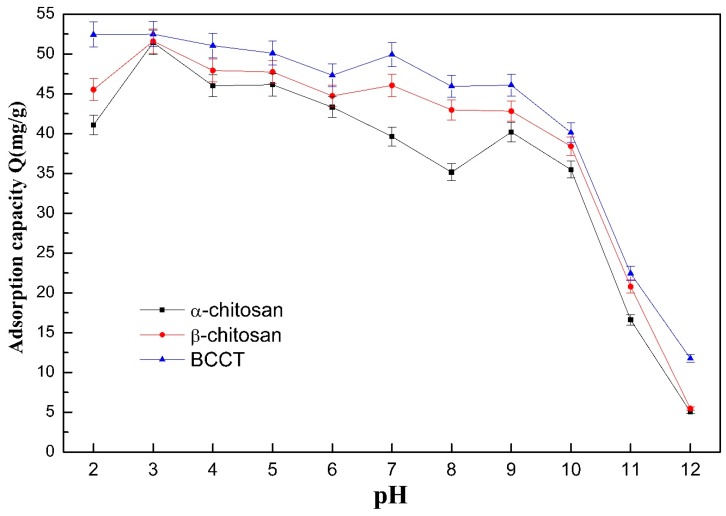
Effects of the pH change on the adsorption capability of the adsorbents.

The dye used in this study was RB221, which possesses sulfonate groups and gives a pH value of about 5.5 as an aqueous solution of 600 ppm concentration. The pH was adjusted (2–12) with NaOH and HCl in concentrations of 2, 1, 0.5, and 0.1 N. The effects of the pH on the adsorption capability of the various sorbents can be seen in Figure 3. The removal of the RB221 dye by the adsorbents, α- and β-chitosan and BCCT, was influenced to a great extent by the pH. The amounts of dye adsorbed/removed per gram of each adsorbent at the strongly acidic condition of pH 2 were 41.07, 45.57, and 52.41 mg/g, respectively. The adsorption capacity of the adsorbents for the dye decreased slowly as the pH changed from 3 to 9 and dropped sharply in the alkaline solutions of pH 10–12. This adsorption behavior was attributed primarily to the Coulomb electrostatic interaction between the anionic sulfonate group (SO_3_^−^) of the dye and the cationic protonated amine group (–NH_3_^+^) of chitosan [27]. The sulfonic salt group (R–SO_3_Na) on the RB221 dye dissociated in the water to form the activated anionic sulfonate (R–SO_3_^−^), as shown in Equation (4). Also, more amine groups on the adsorbent were protonated in a solution of low pH, as shown in Equation (5) [28], increasing the amount of dye molecules adsorbed on the adsorbent. However, an excessive increase in the acidity of the dye solution (pH 2) reduced its adsorption by the adsorbent, as chitosan was over-protonated by hydrogen ions (H^+^) in the strongly acidic environment of low pH, resulting in its degradation by the acid. The extended structure of the modified BCCT adsorbent with polyamination and cross-linking was resistant to acid hydrolysis and had more amine groups, which gave it an enhanced and sustained adsorption capacity under strongly acidic conditions. The modified BCCT was hence stable in its adsorption performance and behaved the best, whereas the unmodified α- and β-chitosan showed a clear decline in adsorption.

(3) $$\mathrm{Dye}–{{\mathrm{SO}}_{3}}^{-}\;+\mathrm{Chitosan}–{{\mathrm{NH}}_{3}}^{+}\overset{\mathrm{Electrostatic}\cdot \mathrm{interactions}}{⇄}\;\mathrm{Dye}–{\mathrm{SO}}_{3^{-}}+\;{{\mathrm{NH}}_{3}}^{+}–\mathrm{Chitosan}$$

(4) $$\mathrm{Dye}–{\mathrm{SO}}_{3}\mathrm{Na}\;\overset{H_{2}O}{\underset{\mathrm{Dissociation}}{⇄}}\;\mathrm{Dye}–{\mathrm{SO}}_{3^{-}}+\;H^{+}$$

(5) $$\mathrm{Chitosan}–{\mathrm{NH}}_{2}\;\overset{H^{+}}{\underset{\mathrm{Pr}\mathrm{otonation}}{⇄}}\;\mathrm{Chitosan}–{{\mathrm{NH}}_{3}}^{+}$$

### 3.2. Discussion of Isothermal Adsorption Models

The isothermal adsorption of the different adsorbents is discussed herein, considering the three types of isotherms commonly used in such cases (i.e., the Langmuir model, Freundlich model, and Temkin model).

#### 3.2.1. Langmuir adsorption isotherm

The Langmuir adsorption isotherm assumes a monolayer coverage on a homogeneous surface and no interaction of the adsorbate with the surrounding substances. The nonlinear and linear representations of this model are given in Equations (6) and (7) below.

(6) $$q_{e}=\frac{Q_{0}bC_{e}}{1+bC_{e}}$$

(7) $$\frac{1}{q_{e}}=\frac{1}{Q_{0}}+\frac{1}{Q_{0}bC_{e}}$$

where *q_e_* is the equilibrium amount of the RB221 dye adsorbed per gram of the sorbent (mg/g), *Q*_0_ is the monolayer adsorption capacity (mg/g), *C_e_* is the equilibrium concentration of the RB221 dye (mg/L), and *B* is the Langmuir adsorption isotherm constant (L/mg).

**Figure 4:**
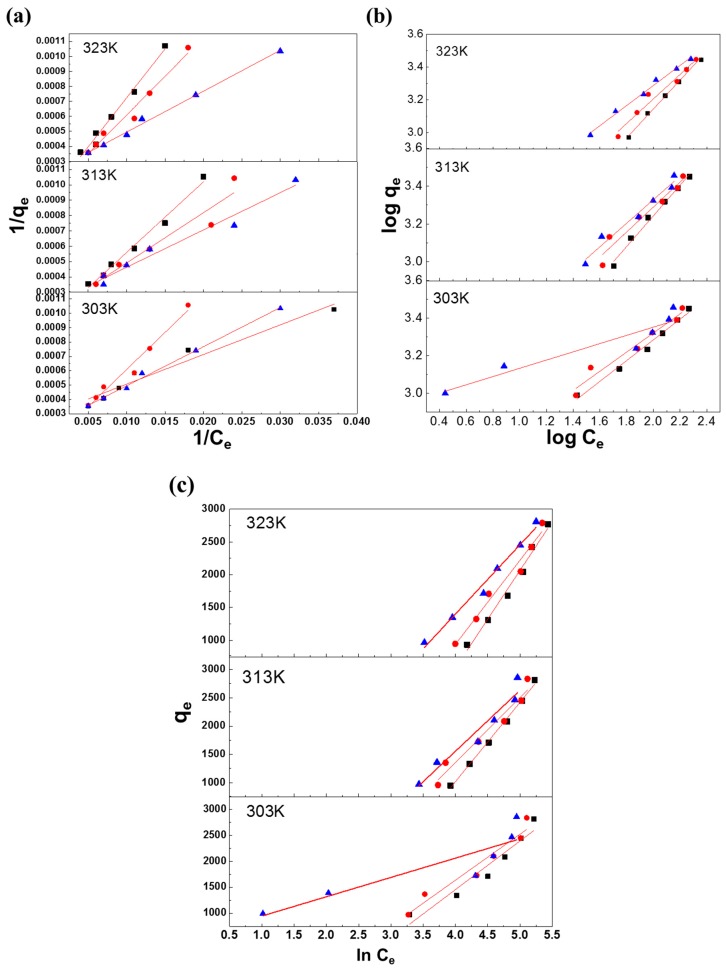
Isothermal adsorption models for the three types of adsorbents (■ α-chitosan, ● β-chitosan, ▲ BCCT) at different temperatures: (**a**) Langmuir isotherm; (**b**) Freundlich isotherm; and (**c**) Temkin isotherm.

A graph of 1/*q_e_* versus 1/*C_e_* was plotted and is shown in Figure 4a. The values of *b* and *Q*_0_ were obtained from the gradient and y-intercept, respectively, which gave the corresponding Langmuir adsorption isotherm constant (L/mg) and the monolayer adsorption capability toward the dye (mg/g). The basic characteristics of the Langmuir isotherm can be expressed by the dimensionless equilibrium constant *R_L_*, which is also known as the separation factor that predicts the nature of the adsorption process.

(8) $$R_{L}=\frac{1}{1+bC_{0}}$$

where *R_L_* is the Langmuir separation factor (L/mg), *B* is the Langmuir adsorption isotherm constant (L/mg), and *C*_0_ is the initial concentration of the RB221 dye (mg/L)s.

The *R_L_* value indicates the nature of the adsorption process: *R_L_* >1 signifies unfavorable adsorption conditions; *R_L_* = 1 represents linear adsorption; 0< *R_L_* <1 occurs when the adsorption conditions are favorable; and *R_L_* = 0 is for irreversible adsorption.

From the data compiled in Table 1, the adsorption modes of the three adsorbents at different temperatures were determined. The results show that the monolayer adsorption capacity *Q*_0_ of the adsorbents, α-chitosan, β-chitosan, and BCCT, of the RB221 dye dropped from 49.01, 54.34, and 625.00 mg/g to 15.31, 20.45, and 36.36 mg/g, respectively as the temperature rose from 303 to 323 K. BCCT thus had the best monolayer adsorption capacity at 303 K and was still more effective than the other two, in this aspect, when the temperature was raised to 323 K.

At different temperatures, the separation factor *R_L_* of the adsorbents, α-chitosan, β-chitosan, and BCCT, for the RB221 dye adsorption was found to decrease from 1.5 × 10^−5^, 1.6 × 10^−5^, and 2.5 × 10^−4^ to 9.2 × 10^−7^, 2.0 × 10^−6^, and 7.3 × 10^−6^, respectively, as the temperature increased from 303 to 323 K. The adsorptions by all the three adsorbents are therefore feasible, with BCCT being the most effective, as more binding sites are available on its surface to which to anchor the dye molecules.

#### 3.2.2. Freundlich Adsorption Isotherm

This model describes the adsorption characteristics at heterogeneous surfaces and is expressed by Equations (9) and (10) below:

(9) $$Q_{e}=K_{f}C_{e}^{\frac{1}{n}}$$

(10) $$\log Q_{e}=\log K_{f}+\frac{1}{n}\log C_{e}$$

where *Q_e_* is the equilibrium amount of the RB221 dye adsorbed per gram of the adsorbent (mg/g), *K_f_* is the Freundlich adsorption isotherm constant (mg/g), *n* is the Freundlich adsorption extent, and *C_e_* is the equilibrium concentration of the RB221 dye (mg/L).

A graph of log *Q_e_* versus log *C_e_* was plotted and is shown in Figure 4b. The values of 1/*n* and *K_f_* were obtained from the gradient and y-intercept, respectively, which gave the corresponding feasibility of the adsorption and the approximate adsorption capacity. *n* > 1 indicates a favorable adsorption.

From the data in Table 1, the approximate adsorption capacities *K_f_* of the three adsorbents of the RB221 dye were compiled. This value decreased from 116.17, 200.45, and 819.03 mg/g to 24.22, 51.49, and 116.17 mg/g for α-chitosan, β-chitosan, and BCCT, respectively, as the temperature rose from 303 to 323 K. BCCT thus possessed the highest approximate adsorption capacity at 303 K and was still superior to the other two in this aspect when the temperature was raised to 323 K. At different temperatures, the adsorption extent n of the adsorbents, α-chitosan, β-chitosan, and BCCT, of the RB221 dye was found to drop from 1.64, 1.96, and 4.56 to 1.14, 1.34, and 1.641, respectively. Adsorptions by all the three adsorbents are therefore feasible, with BCCT being the most effective adsorbent for the RB221 dye.

#### 3.2.3. Temkin Adsorption Isotherm

This model assumes a relationship between the heat of the adsorption and the surface coverage and is expressed in Equation (11):

(11) $$q_{e}=B\ln K_{t}+B\ln C_{e}$$

where *q_e_* is the equilibrium amount of the RB221 dye adsorbed per gram of adsorbent (mg/g)*, K_t_* is the equilibrium binding constant corresponding to the maximum binding energy (dm^3^/g), *B* is a constant related to the heat of sorption (J/mol), and *C_e_* is the equilibrium concentration of the RB221 dye (mg/L).

A graph of *q_e_* versus ln *C_e_* was plotted and is shown in Figure 4c. The values of *B* and *K_t_* were obtained from the gradient and y-intercept, respectively. From the data in Table 1, the equilibrium binding constants *K_t_* corresponding to the maximum binding energy were compiled for the three adsorbents of the RB221 dye. This value decreased from 1.38, 1.59, and 1.91 dm^3^/g to 1.32, 1.36, and 1.45 dm^3^/g for α-chitosan, β-chitosan, and BCCT, respectively, as the temperature rose from 303 to 323 K. BCCT thus had the highest adsorption capacity at 303 K and was still the most effective of the three when the temperature was raised to 323 K. At different temperatures, the heat of adsorption B of the adsorbents, α-chitosan, β-chitosan, and BCCT, of the RB221 dye were found to decrease from 0.87, 1.32, and 4.37 J/mol to 0.50, 0.64, and 0.92 J/mol, respectively. This shows the negative effect exerted by the temperature elevation on the dissipation of the heat during adsorption, which subsequently hindered the dye adsorption by the adsorbents.

### 3.3. Adsorption Thermodynamics

Thermodynamic experiments on the adsorption of RB221 dye by different adsorbents at various temperatures were carried out at pH 5 and an initial dye concentration of 1400 mg/L. The thermodynamic parameters related to the adsorption, such as the Gibbs free energy change (Δ*G*^0^), standard enthalpy change (Δ*H*^0^), and standard entropy change (Δ*S*^0^) were calculated using the van ’t Hoff equation, as shown in Equations (12) and (13) below [26]:

∆G^0^ = −*RT*ln*K*_c_ (12)

(13) $$\ln K_{c}=-\frac{\Delta H^{0}}{RT}+\frac{\Delta S^{0}}{R}$$

where *K_C_* is the distribution constant at different temperatures as given by (*q_e_*/*C_e_*), *C_e_* is the equilibrium concentration of the RB221 dye (mg/L), *q_e_* is the equilibrium amount of the RB221 dye adsorbed per gram of the adsorbent (mg/g), Δ*S*^0^ is the change in the standard entropy (J/mol K), Δ*H*^0^ is the change in the standard enthalpy (KJ/mol), and *R* is the ideal gas constant (8.314 J/mol K).

A graph of ln *K_c_* versus 1000/*T* was plotted. The values of Δ*H*^0^ and *ΔS*^0^*B* were obtained from the gradient and y-intercept, respectively. 

**Figure 5:**
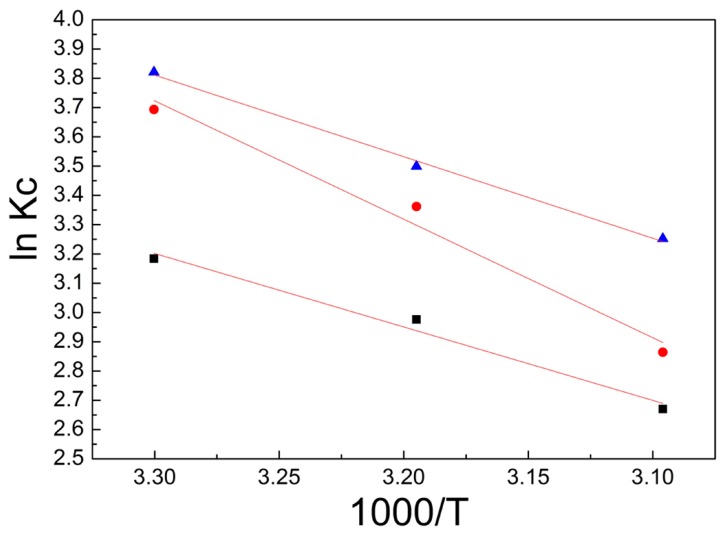
Plot of K_c_ versus 1/T for the determination of the adsorption thermodynamics parameters (■ α-chitosan, ● β-chitosan, and ▲ BCCT).

From the plots in Figure 5 and the calculations performed using the above equations, the results obtained are listed in Table 2. The following conclusions can be drawn.

1. During the adsorption of the RB221 dye in the system by the different adsorbents, as the temperature increased from 303 to 323 K, both the Δ*G*^0^ and Δ*H*^0^ were negative, indicating the spontaneity of the process, as no energy needed to be supplied from the outside.
2. A negative ΔH^0^ showed that the adsorption was an exothermic process. Alkan et al. [27,28,29] noticed a standard enthalpy change of 40–120 KJ/mol caused by chemisorption, which was larger than that by physisorption. In this sense, the enthalpy of the adsorption found in this study is below that of the chemisorption, which indicates that the adsorption in this case may be due to physical interactions.
3. The final concentrations of the RB221 dye removed by the adsorbents at different temperatures were found. It was noticed that the maximum adsorption capacities of α-chitosan, β-chitosan, and BCCT decreased from 1344.29, 1365.99, and 1369.98 mg/g to 1309.34, 1324.48, and 1347.83 mg/g, respectively. This could be attributed to the adsorption of the RB221 dye being exothermic, because of the Coulombic electrostatic force between the sulfonates of the RB221 molecules and the protonated amines on the chitosan. In addition, hydrogen bonding between the adsorbents and dye molecules could be a reason.

### 3.4. Adsorption Kinetics

**Figure 6:**
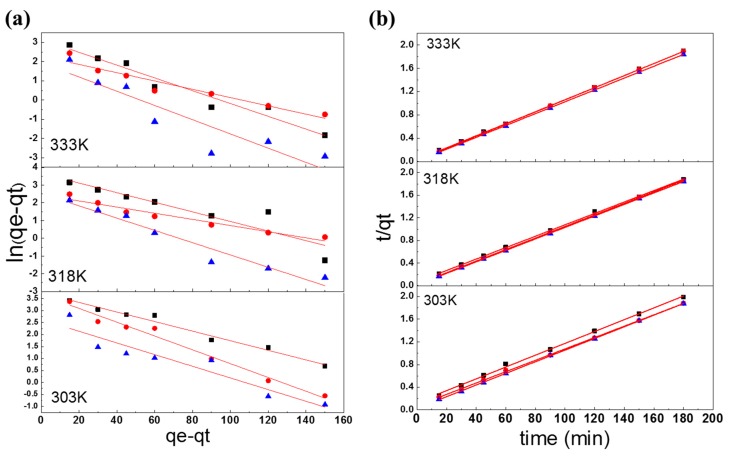

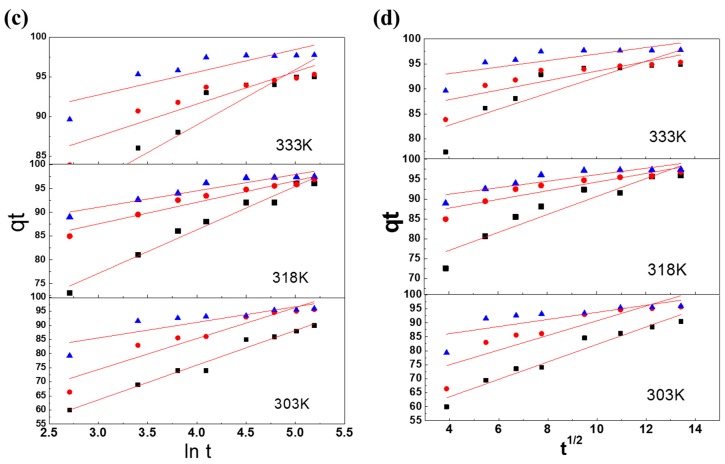
Adsorption kinetics models of the three adsorbents at different temperatures: (**a**) pseudo-first-order model; (**b**) pseudo-second-order model; (**c**) Elovich model; and (**d**) Weber–Morris model (■ α-chitosan, ● β-chitosan, and ▲ BCCT).

In the study of adsorption rates, the two most commonly used models are the pseudo-first-order kinetics and pseudo-second-order kinetics, along with the Elovich model and the Weber–Morris model [26], as shown in Equations (14)–(18). In this section, the adsorption kinetics of the different adsorbents are analyzed and discussed. The adsorption kinetics models of the adsorbents at different temperatures are given in Figure 6.

#### 3.4.1. Pseudo-First-Order Kinetics Model

**Figure 7:**
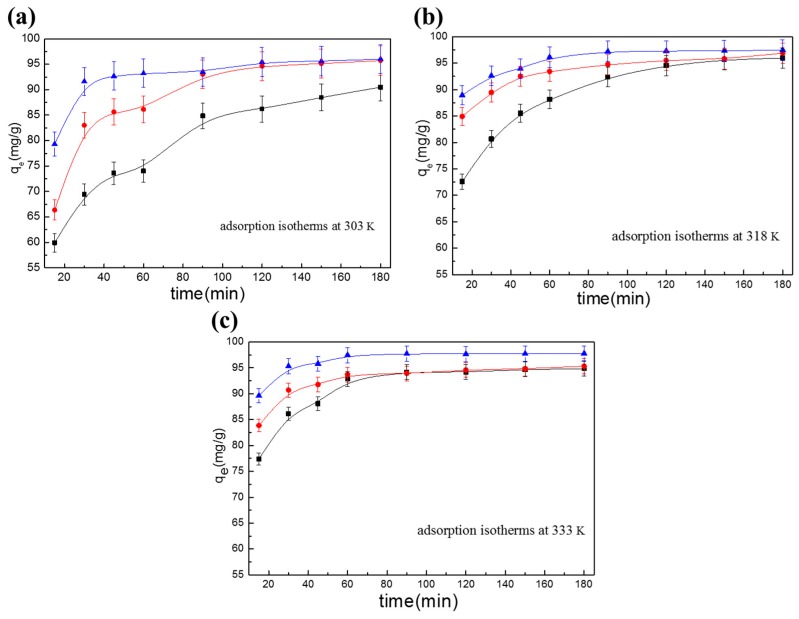
Variations in the adsorption time and adsorption capacity of the adsorbents at different temperatures: (**a**) 303 K; (**b**) 318 K; and (**c**) 333 K (■ α-chitosan, ● β-chitosan, and ▲ BCCT).

Using the pseudo-first-order rate equation, the adsorption kinetics models and behavior of the three adsorbents were obtained, as shown in Table 3 and Figure 7. The pseudo-first-order rate constants *k*_1_ for the RB221 dye adsorption by the adsorbents were found under different temperatures. This value changed from 0.9802, 0.9716, and 0.9760 mg/g to 0.9672, 0.7860, and 0.9636 mg/g for α-chitosan, β-chitosan, and BCCT, respectively, as the temperature varied from 303 to 333 K. Thus, our conclusions are that (1) the temperature rise prevented the adsorption of the dye by the adsorbents and (2) BCCT has the best monolayer adsorption capability under all the temperatures and is capable of the highest adsorption of the three, even at 333 K.

(14) $$\log(q_{e}-q_{t})=\log q_{e}-\frac{k_{1}}{2.303}t$$

where *q_e_* is the equilibrium amount of the RB221 dye adsorbed per gram of the adsorbent (mg/g), *q_t_* is the amount of the RB221 dye adsorbed per gram of the adsorbent at time *t* (mg/g), *k*_1_ is the pseudo-first-order rate constant (1/min), and *t* is the time (min).

#### 3.4.2. Pseudo-Second-Order Kinetics Model

Using the pseudo-second-order rate equation, the adsorption kinetics models and behavior of the three adsorbents were obtained, as shown in Table 3 and Figure 7. *h*, the initial adsorption rates for the RB221 dye were found under different temperatures. This value changed from 96.1538, 100.00, and 97.0874 mg/g min to 97.0874, 96.1538, and 98.0392 mg/g min for α-chitosan, β-chitosan, and BCCT, respectively, as the temperature increased from 303 to 333 K. Hence, the initial adsorption rates of the three adsorbents were similar at different temperatures. k_2_, the pseudo-second-order rate constants for the RB221 dye adsorption were found under different temperatures. This value decreased from 1.6577, 0.9997, and 0.0761 g/mg min to 0.1336, 0.0436, and 0.0114 g/mg min for α-chitosan, β-chitosan, and BCCT, respectively, as the temperature increased from 303 to 333 K. This signified that the initial adsorption was not favored among the three adsorbents at elevated temperatures. A comparison of the *R*^2^ values of the linear regression also shows this model being the more appropriate one to explain the adsorption kinetics of the three adsorbents:

(15) $$\frac{t}{q_{t}}=\frac{1}{k_{2}q_{e}^{2}}+\frac{t}{q_{e}}$$

(16) $$h=k_{2}q_{e}^{2}$$

where *q_e_* is the equilibrium amount of the RB221 dye adsorbed per gram of the adsorbent (mg/g), *q_t_* is the amount of the RB221 dye adsorbed per gram of adsorbent at time *t* (mg/g), *k*_2_ is the pseudo-second-order rate constant (1/min), and *h* is the initial adsorption rate of the adsorbent (mg/g min).

#### 3.4.3. Elovich Model

Using the Elovich rate equation, the adsorption kinetics models and behavior of the three adsorbents were obtained, as shown in Table 3 and Figure 7. α, the initial adsorption rates for the RB221 dye were found under different temperatures. This value changed from 1.01 × 10^2^, 4.74 × 10^2^, and 1.48 × 10^6^ mg/g min to 0.981 × 10^4^, 4.23 × 10^8^, and 1.52 × 10^13^ mg/g min for α-chitosan, β-chitosan, and BCCT, respectively, as the temperature rose from 303 to 333 K. Hence, BCCT had the most prominent initial adsorption rate at 303 K and offered the best adsorption capability, even at 323 K. The desorption constants β for the RB221 dye adsorption were also found under different temperatures. This value changed from 0.0802, 0.0909, and 0.1810 to 0.1470, 0.2452, and 0.3483 for α-chitosan, β-chitosan, and BCCT, respectively, as the temperature increased from 303 to 333 K. This indicated that for all three adsorbents, a large quantity of the dye desorbed during adsorption as the temperature went up, pointing to hydrogen bonding as a second player assisting in the adsorption, in addition to the Coulombic interaction.

(17) $$q_{t}=\frac{1}{\beta }\ln(\alpha \beta )+\frac{1}{\beta }\ln(t)$$

where *q_t_* is the amount of the RB221 dye adsorbed per gram of the adsorbent at time *t* (mg/g), *α* is the initial adsorption rate of the adsorbent (mg/g min), *β* is the desorption constant (g/mg), and *t* is the time (min).

#### 3.4.4. Weber–Morris model

Using the Weber–Morris rate equation, the adsorption kinetics models and behavior of the three adsorbents were obtained, as shown in Table 3 and Figure 7. *k*_dif_, the intraparticle diffusion rate constants for the RB221 dye adsorption were found under different temperatures. This value decreased from 3.1238, 2.6255, and 1.2708 mg/g min to 1.6022, 1.0810, and 0.6522 mg/g min for α-chitosan, β-chitosan, and BCCT, respectively, as the temperature rose from 303 to 333 K. Hence, temperature had an effect on the intraparticle diffusion rate of the adsorbent molecules in the dye. The constants C for the RB221 dye adsorption were also found under different temperatures. This value changed from 51.0390, 64.5030, and 81.0540 to 76.2880, 83.3890, and 90.4380 for α-chitosan, β-chitosan and BCCT, respectively, as the temperature increased from 303 to 333 K. This indicated that the adsorptions by all three adsorbents were influenced by temperature changes. A larger value of C signified a greater impact of adsorbent and dye solution boundary diffusion on the adsorption. BCCT was affected most significantly by the temperature, which could be due to its cross-linked structure, as this made its intraparticle diffusion rate the lowest of the three.

(18) $$q_{t}=k_{\mathrm{dif}}t^{0.5}+C$$

where *q_t_* is the amount of the RB221 dye adsorbed per gram of the adsorbent at time *t* (mg/g), *t* is the time (min), *k_dif_* is the intraparticle diffusion rate constant (mg/g min), and *C* is a constant indicating the influence of adsorbent and liquid phase boundary diffusion on the adsorption. A larger value of C signifies a greater impact of solid and liquid phase boundary diffusion on the adsorption [30,31].

To support these claims, we have summarized the relevant analytical parameters for the dye effluent treatment in Table 4, including the various chitosan-related adsorbents, different types of dye, removal capacity, temperature, and pH for the various chitosan-related nanohybrids [32,33,34,35,36,37,38]. The main contribution of our study is a description of the crosslinking modification of β-chitosan and the successful analysis of its fast adsorption characteristics of Reactive Blue 221 ions in highly acidic environments. The capacity of modified chitosan for adsorbing dye ions improved in acidic environments at a pH of 2–6. Therefore, this study successfully developed an acid-resistant, organic dye-adsorbing material, which is expected to be applied in industrial effluent treatment.

### 3.5. Adsorption Mechanism of BCCT for Reactive Blue 221 Dye at pH 2

#### 3.5.1. Action of Hydrogen Bonding

**Figure 8:**
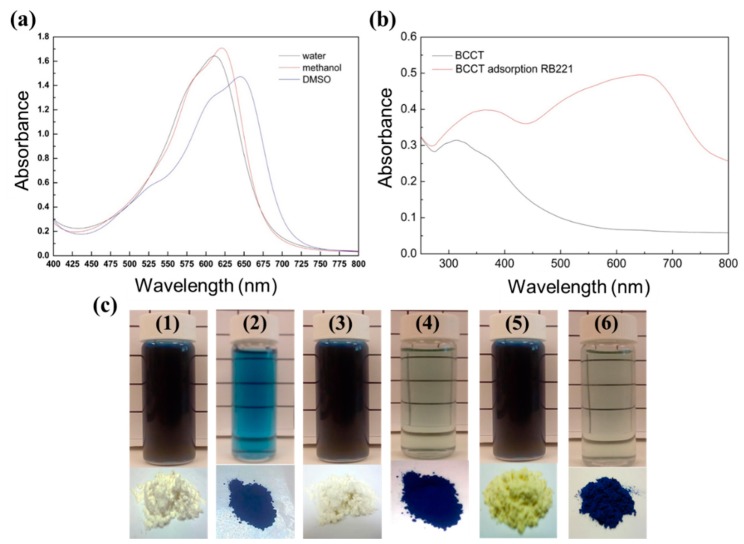
UV–Vis absorption spectroscopy: (**a**) changes in the UV–Vis spectra for the RB221 dye solutions prepared with different solvents (water, methanol, and DMSO) of concentration = 1.14 × 10^−3^ mol/L; and (**b**) UV–Vis spectra of BCCT before and after the dye adsorption. (**c**) Various adsorbents before and after the RB221 dye adsorption in solution: (**1** and **2**) α-chitosan and RB221-adsorbed α-chitosan; (**3** and **4**) β-chitosan and RB221-adsorbed β-chitosan; and (**5** and **6**) BCCT and RB221-adsorbed BCCT.

To evaluate the strength of the hydrogen bonding between the dye and the adsorbent molecules, a number of polar solvents (water, methanol, and DMSO) were selected to determine their interaction with the dye molecules. From Figure 8a, it can be seen that DMSO (UV–VIS λmax = 649.8 nm) is a non-hydrogen-bonding polar solvent. As the electronic energy level π* is more polar than π in polar molecules, a polar solvent reduces the energy of the π→π* transition, resulting in a red-shift of the signal. This contrasts with methanol (λmax = 620.8 nm) and water (λmax = 640 nm), especially water, which is a dispersion medium causing a blue-shift in the UV–Vis signal due to its strong hydrogen-bonding tendency as the RB221 dye dissolved. Based on the changes in the UV–Vis spectra of the BCCT adsorbent before and after the RB221 dye adsorption (Figure 8b), the UV–Vis signal shifted slightly to a longer wavelength after adsorption. This was because the dye molecule possessed a conjugated structure, which absorbed in the ultraviolet region. As more conjugated bonds were introduced into the molecular structure of the adsorbent by the dye adsorption, its absorption wavelength underwent a red-shift. The adsorption of the RB221 dye by BCCT also produced a hyperchromic effect in the latter, indicating a substantial change in its structure [39], consistent with the isothermal adsorption model and adsorption kinetics model obtained in the previous sections. Figure 8c shows the images of the aqueous dye solutions before and after the adsorption of the RB221 dye by α-chitosan, β-chitosan, and BCCT, respectively, as well as the images of the adsorbent/RB221 powder. In addition, Video S1 (see Supplementary Information) shows the RB221 dye adsorption of BCCT for the organic dye ion before and after adsorption. The aqueous solutions appeared to be dark blue before the adsorption of the dye and turned to pale blue after the adsorption. Figure 8c(6) shows the nearly transparent state of the solution after the dye was adsorbed by BCCT, which indicates that BCCT exhibited the best adsorption effect. Also, the powdered adsorbents were all pale yellow before the adsorption but changed exclusively to dark blue after the adsorption.

#### 3.5.2. FTIR Spectroscopy

**Figure 9:**
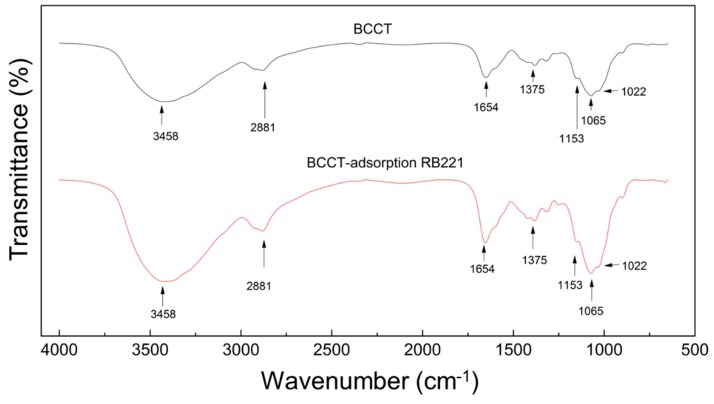
Changes in the FTIR spectra of BCCT before and after the adsorption of the RB221 dye.

Figure 9 shows the changes in the infrared spectra of BCCT before and after the adsorption of the RB221 dye. The characteristic peaks for the primary alcohol and secondary alcohol functional groups on the modified BCCT molecule showed a significant enhancement in intensity after the adsorption of the RB221 dye. The cross-linked structure of the adsorbent was partially degraded in the acidic solution, and hence, the signal strengths of the primary alcohol groups (at a wavenumber of 1022 cm^−1^), secondary alcohol groups (at a wavenumber of 1065 cm^−1^), and glycosidic bonds (at a wavenumber of 1153 cm^−1^) increased. The same also occurred in the amide bonds (at a wavenumber of 2881 cm^−1^) and amine groups (at wavenumbers of 1654 and 3458 cm^−1^), indicating the adsorption of a large amount of dye amine groups on BCCT. This confirmed the adsorption of the RB221 dye by BCCT. The qualitative results obtained from the IR spectra of BCCT before and after the adsorption are listed in Table S1.

#### 3.5.3. Elemental Analysis

The elemental analysis results in Table 5 were used to compare the adsorption capacities of the three adsorbents toward the RB221 dyes. The changes in the sulfur (S) compositions before and after the dye adsorption were found to be 0.33%, 0.34%, and 0.44% for α-chitosan, β-chitosan, and BCCT, respectively, whereas the corresponding nitrogen (N) compositions after dye adsorption were 8.04%, 8.15%, and 8.20%, respectively. The changes in the S/C values (sulfur/carbon ratio) were 0.0079, 0.0082, and 0.0102 for α-chitosan, β-chitosan, and BCCT, respectively. These results showed the ability of BCCT to adsorb and remove the RB221 dye effectively.

#### 3.5.4. Zeta Potential Analysis

From the Zeta potential analysis results, the water of the 600 ppm RB221 dye aqueous solution had a potential of −54.1 mV. This value became −5.64 mV after the decolorization of the solution and adsorption of the dye, which resembled the potential of −5.46 mV of deionized water, indicating the good decolorization and adsorption capacity of BCCT for the RB221 dye.

**Figure 10:**
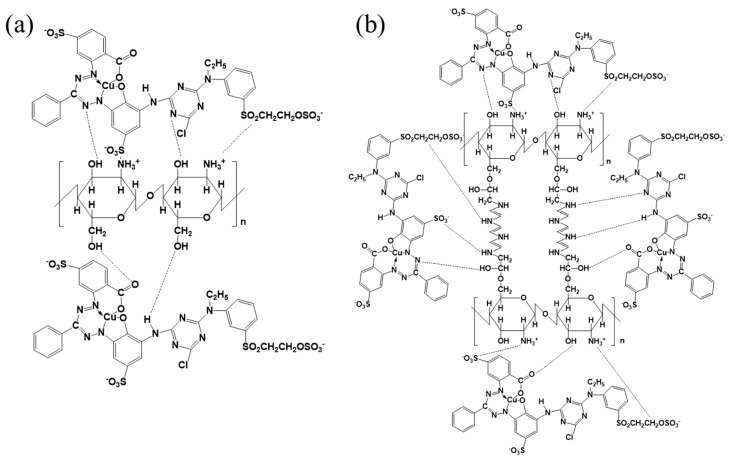
Mechanism of the RB221 adsorption by the adsorbents: (**a**) chitosan adsorbing the RB221 dye; and (**b**) BCCT adsorbing the RB221 dye.

The adsorption mechanisms could be deduced from the above analyses. Figure 10a shows the adsorption of the RB221 dye by the unmodified chitosan, which was achieved primarily through the lone pairs on the hydroxyl group and amine group of the adsorbent molecule. Coulombic electrostatic interactions involving the anionic sulfonate group (SO_3_^−^) of RB221 and the cationic protonated amine group (–NH_3_^+^) of chitosan and hydrogen bonding captured the RB221 dye molecules and removed them from the solution. The adsorption mechanism of the cross-linked and modified BCCT is given in Figure 10b. Aside from the attraction of the RB221 dye due to the chitosan main chain, more lone pairs were present on the amine groups of the polyamines grafted as the cross-linkers in BCCT. These lone pairs enhanced the aforementioned adsorption mechanism for the RB221 dye. The hydrogen bonding and Coulomb electrostatic interactions described herein agreed with the results of the earlier analyses (UV–Vis, FTIR spectroscopy, elemental analysis, Zeta potential analysis, etc.) [40,41,42].

## 4. Conclusions

In this study, β-chitosan was used as the main raw material for the grafting and crosslinking reactions. The capture, adsorption, and removal capacity of various types of chitosan adsorbents toward the RB221 dye was tested and compared. The experiments were performed on the adsorption of the RB221 dye by unmodified α- and β-chitosan and cross-linkage–modified BCCT, at different temperatures and for different durations, along with the adsorption kinetics calculations. According to the results, as the temperature increased from 303 to 333 K, the initial adsorption rates of the adsorbents, α-chitosan, β-chitosan, and BCCT, for the RB221 dye changed from 1.01 × 10^2^, 4.74 × 10^2^, and 1.48 × 10^6^ mg/g min to 5.98 × 10^4^, 4.23 × 10^8^, and 1.52 × 10^13^ mg/g min, respectively. BCCT showed the best adsorption capability for the dyes at all temperatures. These results confirmed the successful introduction of a polyaminated and cross-linked extended structure as a modification for the BCCT adsorbent, which makes it resistant to acid hydrolysis and gives it the functional amine group for dye adsorption, thereby promoting the adsorption capability of BCCT under strongly acidic conditions. The compound synthesized in this study is expected to help the adsorption of anionic organic dyes in acidic effluent and to achieve dye effluent purification in a relatively short time.

## Figures and Tables

**Table 1:** The isotherms and parameters for Reactive Blue 221 dye adsorption at different temperatures and by different adsorbents (tested at pH = 5 and *t* = 24 h).

Adsorbent Type	α-Chitosan			β-Chitosan			BCCT		
Temperature (K)	303	313	323	303	313	323	303	313	323
Langmuir isotherm									
*Q*_0_ (mg/g)	49.02	21.14	15.31	54.35	31.45	20.45	625.00	41.49	36.36
*b* (L/mg)	68.00	525.56	1088.33	61.33	159.00	489.00	4.00	120.50	137.50
*R*_L_	1.5 × 10^−5^	1.9 × 10^−6^	9.2 × 10^−7^	1.6 × 10^−5^	6.3 × 10^−6^	2.0 × 10^−6^	2.5 × 10^−4^	8.3 × 10^−6^	7.3 × 10^−6^
*R* ^2^	0.9552	0.9917	0.9972	0.9515	0.9210	0.9749	0.8922	0.9676	0.9954
Freundlich isotherm									
*N*	1.64	1.23	1.14	1.96	1.52	1.34	4.56	1.61	1.64
1/*n*	0.61	0.81	0.88	0.51	0.66	0.75	0.22	0.62	0.61
*k*_f_ (mg/g)	116.17	41.91	24.22	200.45	93.78	51.49	819.03	121.53	116.17
*R* ^2^	0.9886	0.9893	0.9939	0.9613	0.9472	0.9675	0.8936	0.9688	0.9925
Temkin isotherm									
*K*_t_ (dm^3^/g)	1.38	1.36	1.32	1.59	1.43	1.36	1.91	1.48	1.45
*b* (J/mol)	0.87	0.57	0.50	1.32	0.80	0.64	4.37	0.95	0.92
*R* ^2^	0.9854	0.995	0.9837	0.9485	0.9569	0.9729	0.8131	0.9416	0.9854

**Table 2:** Adsorption thermodynamic parameters of the adsorbents of the Reactive Blue 221 dye (tested at pH 5 and an initial dye concentration of 1400 mg/L).

Adsorbent Type	Final Concentration *C*_e_ (mg/L)	*T* (K)	Final Dye Removal *C*_Ae_ (mg/L)	*K*c	Thermodynamic Parameter		
					ΔG^0^ (KJ/mol)	ΔH^0^ (KJ/mol)	ΔS^0^ (J/mol k)
α-chitosan	55.70	303	1344.29	24.133	−8.020	−20.837	42.145
	67.92	313	1332.07	19.610	−7.745		
	90.65	323	1309.34	14.443	−7.171		
β-chitosan	34.00	303	1365.99	40.172	−9.304	−33.633	80.038
	46.90	313	1353.09	28.848	−8.749		
	75.51	323	1324.48	17.539	−7.692		
BCCT	30.01	303	1369.98	45.643	−9.625	−23.174	44.792
	41.09	313	1358.91	33.072	−9.105		
	52.16	323	1347.83	25.838	−8.733		

**Table 3:** Kinetic models of the adsorbents for the Reactive Blue 221 dye adsorption.

Adsorbent Type	α-Chitosan			β-Chitosan			BCCT		
Temperature (K)	303	318	333	303	318	333	303	318	333
Pseudo-first-order model									
*k*_1_ (1/min)	0.9802	0.9734	0.9672	0.9716	0.9828	0.9786	0.9760	0.9660	0.9636
*R* ^2^	0.9796	0.8496	0.9519	0.9812	0.9514	0.9263	0.9027	0.9503	0.8372
Pseudo-second-order-model									
*h* = *k*_2_ × *q*_e_^2^ (mg/g min)	96.1538	99.0099	97.0874	97.0874	98.0392	96.1538	100.0000	98.0392	98.0392
*k*_2_ (g/mg min)	1.6577	0.4755	0.1336	0.9997	0.0769	0.0436	0.0761	0.0297	0.0114
*R* ^2^	0.9981	0.9994	0.9999	0.9997	0.9999	1.0000	0.9999	1.0000	1.0000
Elovich model									
α (mg/g min)	1.01 × 10^2^	1.84 × 10^3^	5.98 × 10^4^	4.74 × 10^2^	5.65 × 10^7^	4.23 × 10^8^	1.48 × 10^6^	5.12 × 10^10^	1.52 × 10^13^
β (g/mg)	0.0802	0.1080	0.1470	0.0909	0.2209	0.2452	0.1810	0.2903	0.3483
*R* ^2^	0.9795	0.9713	0.8883	0.9100	0.9500	0.8537	0.7583	0.9162	0.7645
Weber–Morris model									
*k*_dif_ (mg/g min)	3.1238	2.2639	1.6022	2.6255	2.2639	1.0810	1.2708	0.8187	0.6522
*C*	51.0390	68.0210	76.2880	64.5030	68.0210	83.3890	81.0540	87.9630	90.4380
*R* ^2^	0.9519	0.8985	0.7625	0.8036	0.8985	0.8529	0.6214	0.8012	0.6110

**Table 4:** Summary of the application of various chitosan-related nanohybrids in the dye effluent treatment.

Adsorbents	Dye	Adsorption Capacity *Q*_max_ (mg/g)	Temperature (°C)	pH	Reference
Chitosan beads	Reactive yellow	9	50	2	[32]
	Reactive blue	10	25	2	
	Reactive red	7.5	50	2	
Cross-linked chitosan	Direct blue 71	14	--	4	[33]
	Acid black 1	37	--	4	
Glutaraldehyde-cross-linked chitosan (GLA-CTS)	Methyl green	33.7	25	4	[34]
Cross-linked chitosan	Indigo carmine	0.6	35	4	[35]
Magnetic γ-Fe_2_O_3_/crosslinked chitosan composite	Methyl orange	29.8	25	4	[36]
Zinc oxide nanoparticles/chitosan	Direct blue 78	34.5	21	2	[37]
Nano-ZnO/chitosan composite beads (Nano-ZnO/CT-CB)	Reactive black 5	189.4	40	4	[38]
β-chitosan cross-linked with Triethylenetetramine (BCCT)	Reactive blue 221	52.4	25	2	Our work
		625.00 ^a^	30	5	

^a^ The isotherms and parameters for the Reactive Blue 221 dye adsorption by BCCT adsorbent (tested at pH = 5 and *t* = 24 h).

**Table 5:** Elemental analyses on the adsorbents before and after their adsorption of the RB221 dye.

Sample	Element (%)						
	C	N	H	O	S	Total	S/C
α-chitosan	41.85	7.60	7.99	40.79	-	98.24	-
β-chitosan	41.72	7.77	8.06	40.63	-	98.18	-
BCCT	42.06	7.78	8.08	40.40	-	98.32	-
α-chitosan adsorption of RB221	41.34	8.04	7.05	41.27	0.33	98.03	0.0079
β-chitosan adsorption of RB221	41.45	8.15	7.07	41.55	0.34	98.55	0.0082
BCCT adsorption of RB221	42.98	8.20	6.98	40.04	0.44	98.63	0.0102

## Supplementary Materials

The following are available online at http://www.mdpi.com/2073-4360/10/12/1328/s1: Figure S1: FTIR analysis on chitosan, β-chitosan crosslinked with triethylenetetramine, and its intermediate products; Figure S2: ^13^C solid-state NMR of chitosan and β-chitosan crosslinked with triethylenetetramine; Table S1: Characterization of the functional groups on the adsorbent before and after RB221 adsorption, using infrared spectroscopy analysis; and Video S1: Demonstration of the RB221 dye adsorption of BCCT for dye anion before and after adsorption.
