# Supplementary material for: Isothermal Adsorption Properties for the Adsorption and Removal of Reactive Blue 221 Dye from Aqueous Solutions by Cross-Linked β-Chitosan Glycan as Acid-Resistant Adsorbent
Source: Polymers (Basel). 2018 Nov 30;10(12):1328. doi: 10.3390/polym10121328 (PMC6401849; doi:10.3390/polym10121328)
Supplement: Supplementary file 1 [file polymers-10-01328-s001.zip › polymers-387037.si.proofdone/polymers-387037-supplementary/Chiu-3 Supporting Information (polymers-387037) 27-Nov-2018.docx]

**Supporting Information**

Isothermal Adsorption Properties for the Adsorption and Removal of Reactive Blue 221 Dye from Aqueous Solutions by Cross-Linked β-Chitosan Glycan as Acid-Resistant Adsorbent

Chih-Wei Chiu ^1,*^, Ming-Tsung Wu ^1^, Jimmy Chi-Min Lee ^2^ and Ting-Yu Cheng ^1^

^1^ Department of Materials Science and Engineering, National Taiwan University of Science and Technology, Taipei 10607, Taiwan

^2^ Clean Instruments Co., Ltd., New Taipei City 24301, Taiwan

*Corresponding author:

Tel: +886-2-2737-6521; Fax: +886-2-2737-6544; E-mail: [cwchiu@mail.ntust.edu.tw](mailto:cwchiu@mail.ntust.edu.tw) (C.-W. Chiu)


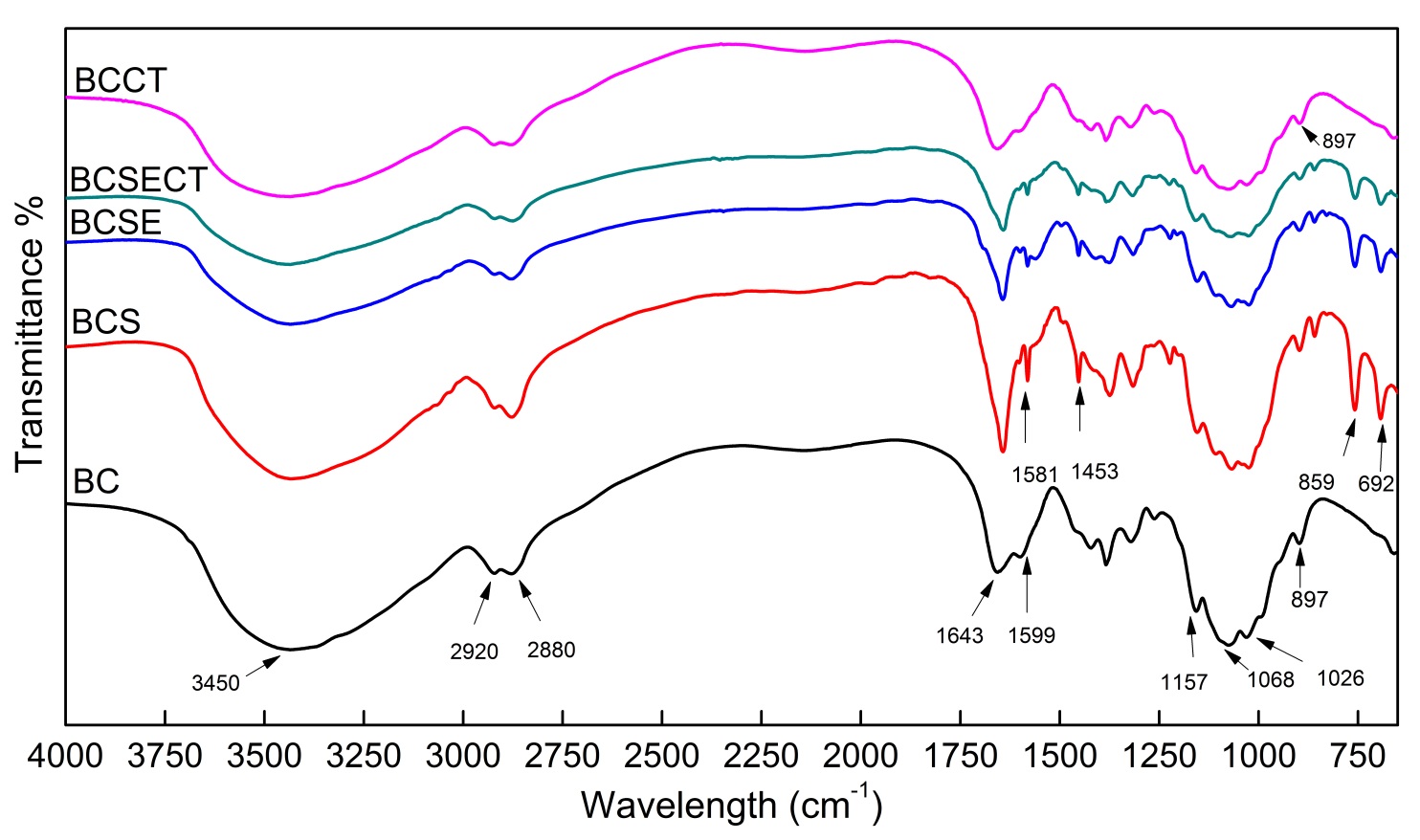


**Figure S1.** FTIR analysis on chitosan, β-chitosan crosslinked with triethylenetetramine, and its intermediate products.


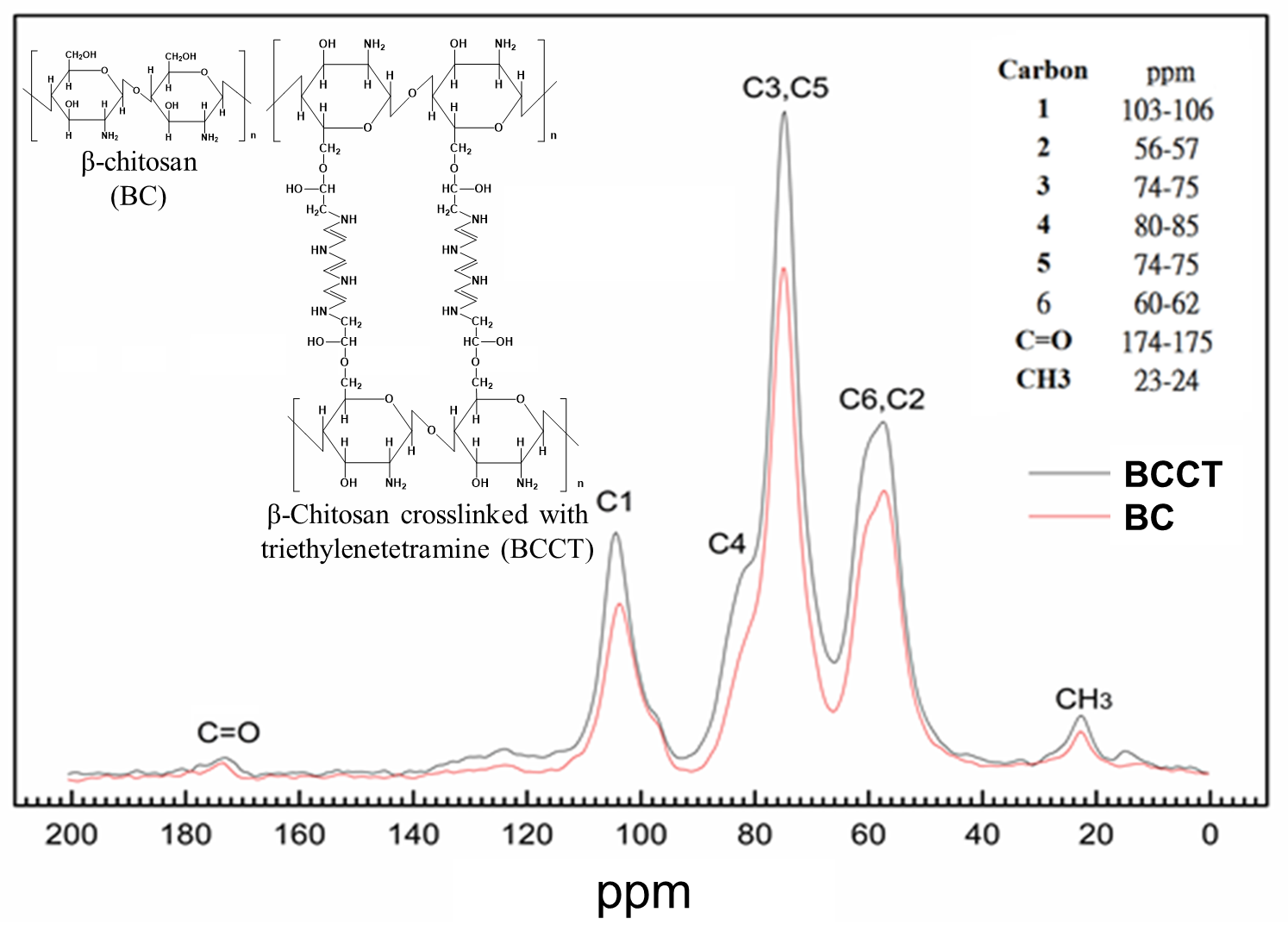


**Figure S2.** ^13^C solid-state NMR of chitosan and β-chitosan crosslinked with triethylenetetramine.

**Table S1.** Characterization of the functional groups on the adsorbent before and after RB221 adsorption, using infrared spectroscopy analysis.

| Frequency peaks (cm^-1^) | | Functional group |
| --- | --- | --- |
| BCCT | RB221-adsorbed BCCT |  |
| 1022 | 1022 | Primary alcohol |
| 1065 | 1065 | Secondary alcohol |
| 1153 | 1153 | Glycosidic bond |
| 1375 | 1375 | Carboxylic acid |
| 1654 | 1654 | Amide group |
| 2881 | 2881 | C-H |
| 3458 | 3458 | -OH , -NH_2_ |
